# *Weissella*: From Beneficial Probiotics to Opportunistic Pathogens—A Review

**DOI:** 10.3390/nu17193162

**Published:** 2025-10-07

**Authors:** Weiqing Ma, Xiang Liu, Yadi Jing, Meixia Zhang, Xin Zhang, Changfa Wang, Muhammad Zahoor Khan, Mingxia Zhu

**Affiliations:** 1 College of Agriculture and Biology, Liaocheng University, Liaocheng 252000, China; 2 Jining Animal Disease Prevention and Control Center, Jining 272100, China

**Keywords:** *Weissella*, probiotics, metabolism, disease, characteristic

## Abstract

*Weissella*, a genus of Gram-positive, facultatively anaerobic lactic acid bacteria, has emerged as a significant component of human microbiota with diverse biotechnological and therapeutic applications. This narrative review examines the current state of knowledge regarding *Weissella* taxonomy, physiological characteristics, and functional properties based on research spanning from 1993 to present. *Weissella* species demonstrate remarkable versatility, producing bioactive metabolites including exopolysaccharides (EPS), bacteriocins, and organic acids that confer antimicrobial, antioxidant, and anti-inflammatory properties. These bacteria show significant potential in food fermentation, probiotic applications, and therapeutic interventions for gut health, obesity, and inflammatory conditions. However, challenges persist regarding strain-specific pathogenicity, particularly with *W. confusa* as an opportunistic pathogen, and the need for comprehensive safety evaluations. Current limitations include variability in probiotic efficacy, incomplete understanding of host-microbe interactions, and gaps in metabolic pathway characterization. This review provides a foundation for advancing *Weissella* research and applications while highlighting critical areas requiring further investigation to fully harness their biotechnological and therapeutic potential.

## 1. Introduction

The human microbiome comprises a complex ecosystem of microorganisms, predominantly represented by the bacterial phyla Bacillota, Bacteroidetes, Proteobacteria, and Actinobacteria, which collectively maintain dynamic homeostasis with the host under physiological conditions [1,2]. Within this intricate microbial landscape, the genus *Weissella*, a significant member of the Bacillota phylum, has emerged as a microorganism of considerable interest due to its diverse physiological functions and potential therapeutic applications.

Originally classified within the genus *Leuconostoc*, *Weissella* was established as a distinct genus in 1993 following comprehensive phylogenetic analyses that revealed its unique taxonomic identity [3]. Morphologically, *Weissella* species are characterized as Gram-positive bacteria exhibiting pleomorphic cell shapes ranging from short rods to cocci, demonstrating remarkable environmental adaptability [4]. The genus displays ubiquitous ecological distribution, inhabiting diverse environments including soil, aquatic systems, plant surfaces, and the gastrointestinal tracts of humans and animals, where it constitutes an integral component of the gut microbiota.

The multifaceted roles of *Weissella* extend beyond mere colonization, encompassing significant contributions to host physiology through both direct bacterial effects and indirect metabolite-mediated mechanisms. Clinical investigations have demonstrated the immunomodulatory potential of *Weissella* species, exemplified by *W. cibaria* JW15, which significantly enhanced Natural Killer cell activity following daily administration of 1 × 10^10^ CFU to human subjects [5]. Recent advances have revealed novel applications for *Weissella*-derived products, including bacterial ghosts generated through sodium hydroxide treatment, which effectively stimulate macrophage activation and upregulate pro-inflammatory cytokine expression (*IL-1β*, *TNF-α*, and *iNOS*), suggesting potential as immunotherapeutic agents [6]. Furthermore, certain *Weissella* strains demonstrate antimicrobial properties through bacteriocin production, with *W. hellenica* QU 13 synthesizing broad-spectrum antibacterial compounds that lack typical N-terminal signal sequences [7].

These beneficial characteristics have positioned *Weissella* as a promising candidate for probiotic and biotechnological applications, particularly in food fermentation and biopharmaceutical development [8,9]. However, the clinical significance of *Weissella* is complicated by reports of opportunistic pathogenicity, with *W. conflusa* identified as a causative agent of neonatal sepsis in veterinary settings [10]. This dual nature underscores the complexity of host-microbe interactions and highlights the necessity for comprehensive strain-level characterization. Despite substantial progress in *Weissella* research, several critical knowledge gaps persist, including challenges in accurate strain identification, variability in probiotic efficacy across different strains, and incomplete understanding of the molecular mechanisms governing host-microbe interactions. These limitations impede the translation of research findings into practical applications and clinical interventions.

This comprehensive narrative review aims to synthesize current knowledge regarding the taxonomy, physiological characteristics, biotechnological potential, and health implications of *Weissella* species. Through systematic analysis of research published in the Web of Science database since the genus establishment in 1993, we provide an evidence-based foundation for advancing *Weissella* research and applications, while identifying future research directions essential for realizing its full potential in human health promotion and industrial innovation.

## 2. Taxonomy and Functional Characteristics of *Weissella*

Prior to 1993, *Weissella* species were classified within the genus Lactobacillus. The establishment of *Weissella* as a distinct genus was initiated following the seminal work of Collins et al., who demonstrated discernible differences between *Weissella* and *Leuconostoc* based on comprehensive phenotypic, biochemical, and 16S rRNA sequence analyses, particularly within the V6 hypervariable region (positions 1007–1022) [3]. This reclassification marked the beginning of a new era in *Weissella* research and taxonomy.

Building upon this foundational work, recent phylogenetic studies have significantly advanced our understanding of *Weissella* taxonomy through comprehensive genomic analyses. Based on Average Nucleotide Identity, Average Amino Acid Identity, 16S rRNA gene sequences, and homology analysis between 16S rRNA genes and complete genomes, several species have been confirmed as distinct taxonomic entities. These include *W. beninensis* LMG 25373, *W. fabalis* LMG 26217, *W. uvarum* B18NM42, *W. fabaria* LMG 24289, *W. diestrammenae* DSM 27840, and *W. ghanensis* DSM 19935, with each strain representing individual species within the genus [11]. *Weissella* comprises a genus of Gram-positive, facultatively anaerobic, lactic acid-producing bacteria that exhibit wide ecological distribution across diverse environments. These microorganisms are commonly found in host organisms, feces, soil, and fermented plant-based foods (Table 1), with the genus encompassing several well-characterized species, including *W. cibaria*, *W. confusa*, *W. kimchi*, *W. sagaensis*, and other ecologically significant strains. However, the clinical significance of this genus extends beyond its ecological distribution. While most *Weissella* strains are recognized for their probiotic properties, certain species exhibit opportunistic pathogenic behavior under specific conditions. *W. confusa* represents the most clinically significant species within this genus, having been isolated from various clinical cases including bacteremia, endocarditis, postoperative osteomyelitis, sepsis, and more recently, meningitis [12]. The pathogenicity of *W. confusa* is primarily attributed to the production of heat-stable exopolysaccharides (EPSs), which are considered potential virulence factors [13]. Interestingly, host-specific interactions appear to modulate pathogenic expression significantly. For instance, *W. confusa* strains isolated from locusts demonstrate reduced virulence potential, lacking toxin production and secretion systems, suggesting strain-specific genomic adaptations to particular host niches [14]. These findings underscore the critical importance of source-dependent safety evaluations for *Weissella* applications in food and therapeutic contexts.

Paradoxically, despite pathogenic concerns with certain strains, *Weissella* species demonstrate considerable beneficial properties with significant industrial potential. Glucans derived from *W. confusa* exhibit remarkable functional characteristics, including excellent hydrophilicity, thermal stability, and heavy metal chelation activity, supporting their application as functional additives in food processing and environmental remediation strategies [15]. Similarly, α-D-glucans synthesized by *W. cibaria* PDER21 demonstrate superior antioxidant properties, enhanced water-binding capacity, and improved solubility characteristics, thereby expanding their utility in diverse food processing applications [16]. These bioactive compounds represent promising candidates for the development of functional food ingredients and nutraceutical products. Furthermore, the probiotic potential of *Weissella* species is evidenced by their ability to promote the growth of beneficial bacterial populations through specific mechanisms. In vitro studies have demonstrated that *Weissella* strains can significantly enhance the proliferation of beneficial bacteria, including *Lactobacillus* spp. and *Bifidobacterium* spp., primarily through EPS production and associated prebiotic effects [17]. This symbiotic relationship highlights the potential of *Weissella* as a probiotic adjuvant in maintaining gut microbiota homeostasis and supporting digestive health.

**Table 1:** Common species and sources of *Weissella*.

Strain	Source	References
*W. ceti*	Beaked whales (*Mesoplodon bidens*)	[18]
*W. cibaria*	Sorghum	[19]
*W. cibaria* GM93m3	Raw goat milk	[20]
*W. cibaria strain* CXO-01	Saliva	[21]
*W. confusa*	Feces	[22]
*W. diestrammenae*	Gut (*Diestrammena coreana*)	[23]
*W. fabaria*	Cocoa fermentation	[24]
*W. fangxianensis*	Chinese rice wine starter	[25]
*W. fermenti*	Kimchi	[26]
*W. jogaejeotgali*	Korean jogae jeotgal (*Fermented clams*)	[27]
*W. kimchii*	Green onion	[28]
*W. muntiaci*	Faece (*Muntiacus reevesi*)	[29]
*W. oryzae*	Fermented rice grains	[30]
*W. sagaensis*	Traditional Chinese yogurt	[31]
*W. soli*	Soil	[32]

## 3. Growth Conditions and Physiological Characteristics

*Weissella* species exhibit distinctive morphological and physiological characteristics that define their growth requirements and metabolic capabilities. Colonies present as small, circular, gray-white formations with a characteristic moist appearance. Microscopically, *Weissella* cells are short, rod-shaped bacteria that typically occur in pairs or short chains [33]. These bacteria demonstrate species-specific growth optima, although most strains favor mildly acidic conditions with pH ranges of 5.0–6.5 and temperature ranges of 30–37 °C. Growth becomes significantly inhibited under neutral to alkaline conditions, resulting in reduced biomass accumulation and extended lag phases [34]. *Weissella* species are heterotrophic and facultatively anaerobic, enabling survival in diverse oxygen environments.

*Weissella* species utilize various carbohydrates including glucose, maltose, and sucrose as primary carbon sources. Through heterofermentative pathways, particularly the phosphoketolase pathway, these bacteria generate multiple bioactive metabolites including lactic acid, bacteriocins, hydrogen peroxide, exopolysaccharides (EPSs), and diacetyl [35,36,37]. W. viridescens demonstrates additional metabolic versatility by producing volatile organic acids (acetic, propionic, and butyric acids), esters, and free amino acids during fermentation processes. The concentrations of three sweet amino acids—threonine, serine, and glycine—increase proportionally with salt concentration, contributing to both flavor development and preservation properties in food systems [38,39].

Recent metabolomic investigations have revealed remarkable stress tolerance capabilities in *Weissella* species. *W. confusa* demonstrates exceptional salinity tolerance, surviving concentrations up to 35% NaCl in MRS medium for 24 h. This tolerance is mediated through the accumulation of osmoprotectants such as ectoine, lactose metabolism alterations, and modified amino acid and nucleotide metabolic pathways [40]. Under combined acid and bile salt stress conditions, *W. confusa* ZJU exhibits significant metabolic reprogramming, including upregulation of alanine, aspartate, and glutamate metabolism pathways. Enhanced glycolysis and gluconeogenesis pathways serve as central mechanisms for carbohydrate metabolism, conferring increased tolerance to bile salt stress [41].

EPS production in *Weissella* species has been systematically optimized using Response Surface Methodology (RSM). For *W. confusa* 126, optimal cultivation parameters were determined as: cultivation time of 48.50 h, sucrose concentration of 24.00 g/L, pH 7.00, and yeast extract concentration of 2.50%. Under these optimized conditions, EPS production reached 3.00 g/L [42]. Comparative analysis between wild-type and mutant strains revealed minimal variation in EPS production capacity, with yields ranging from 5490.16 to 5580.72 mg/L [43].

## 4. Microbial Regulation Mechanisms of *Weissella*

**Figure 1:**
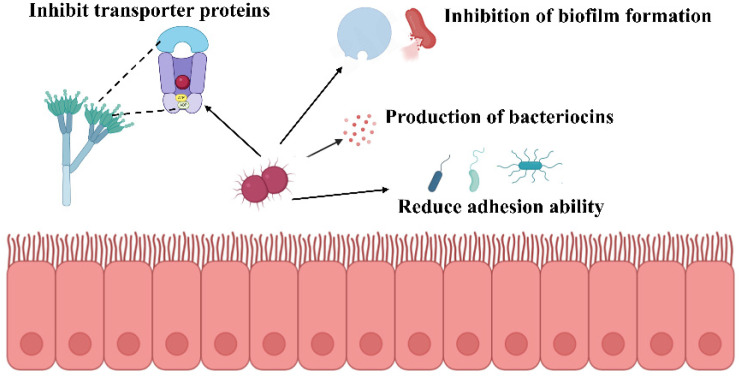
Potential antibacterial mechanisms of *Weissella*.

*Weissella* species demonstrate substantial antimicrobial potential through diverse biological mechanisms involving synergistic metabolite interactions and multiple inhibitory pathways (Figure 1). These lactic acid bacteria exhibit broad-spectrum activity against both bacterial and fungal pathogens through distinct yet complementary mechanisms of action. The antimicrobial efficacy of *Weissella* species has been extensively documented against various pathogenic bacteria. *W. cibaria* JW15 demonstrates inhibitory activity against multiple foodborne pathogens, including Listeria monocytogenes, Salmonella typhimurium, Salmonella enteritidis, and Escherichia coli. Beyond direct antimicrobial effects, this strain interferes with pathogen adhesion to intestinal epithelial cells, thereby providing protective benefits to the intestinal barrier. Additionally, *W. cibaria* JW15 exhibits significant antioxidant properties through the scavenging of reactive oxygen species, including DPPH, ABTS, and hydroxyl radicals, consequently preventing lipid peroxidation processes [44]. Mechanistic studies have elucidated specific pathways through which *Weissella* species exert antimicrobial effects. Kibar et al. investigated the impact of *Weissella*-derived compounds on Streptococcus mutans survival and biofilm formation through controlled in vitro experiments. Their findings demonstrated that glucan at 50 mg/mL concentration inhibited biofilm formation by 70%, with antioxidant activity exhibiting dose-dependent responses [45]. The cell-free culture supernatant of *W. confusa* WM36 contains multiple antimicrobial compounds, primarily organic acids (lactic and acetic acids) and 2,4-di-tert-butylphenol. These metabolites demonstrate concentration-dependent antimicrobial activity against Salmonella typhi. At 40% concentration, the supernatant achieved greater than 99.99% inhibition of biofilm formation, while 20% concentration completely suppressed bacterial motility. Furthermore, at 10% concentration, these metabolites downregulated critical virulence genes associated with type III secretion systems, effector proteins, and quorum-sensing mechanisms [46].

Bacteriocin production represents another significant antimicrobial mechanism in *Weissella* species. These ribosomally synthesized antimicrobial proteins or peptides are encoded by bacterial genes and provide competitive advantages in natural microbial environments. In *W. confusa* MBF8-1, a 17,643 bp plasmid designated pWcMBF8-1 governs bacteriocin production. This genetic element encodes double-glycine motif peptides (Bac1, Bac2, Bac3), an ABC transporter complex (BacTE), and a putative immunity protein (BacI), enabling the strain to survive in highly competitive microbial ecosystems [47]. *Weissella* species also exhibit notable antifungal properties through distinct mechanisms. *W. confusa* AL3, isolated from human gut microbiota, demonstrates inhibitory activity against Aspergillus flavus MTCC 2798. Chemical analysis of the methanol extract identified cyclo-leucyl-proline and 1,2-benzenedicarboxylic acid as the primary antifungal compounds responsible for this activity [48]. Similarly, the cell-free supernatant of *W. cibaria* BYL4.2 exhibits pH-dependent antifungal activity that remains stable following protein treatment. Metabolomic analysis using liquid chromatography-mass spectrometry identified D-tartaric acid as a key antifungal metabolite that inhibits Penicillium chrysogenum growth through disruption of ABC transporter metabolic pathways [49].The antimicrobial activity of *Weissella* species operates through multiple coordinated mechanisms, encompassing pathogen growth inhibition, adhesion interference, reactive oxygen species scavenging, antimicrobial metabolite production, and fungal growth suppression. The synergistic interactions among these mechanisms underscore the considerable potential of *Weissella* species in antimicrobial and antioxidant applications, providing substantial theoretical foundations and practical frameworks for developing novel biotherapeutic agents.

## 5. Potential of *Weissella* in the Food Industry

Probiotics are defined as live microorganisms that confer health benefits to the host through multiple mechanisms, including adherence to host tissues, immune function enhancement, modulation of host metabolic activities, and performance of beneficial metabolic functions [50]. *Weissella* species have emerged as promising probiotic candidates due to their exceptional physiological characteristics, including superior environmental tolerance, antimicrobial activity, β-glucosidase activity, and intestinal adhesion capabilities. Among these attributes, the remarkable ability of *Weissella* species to produce exopolysaccharides represents a particularly valuable trait for industrial applications.

The exopolysaccharides produced by *Weissella* species possess distinctive adhesive, cohesive, antioxidant, and immunomodulatory properties that confer significant value in both food additive and biomedical applications. *W. confusa* demonstrates exceptional utility in sourdough fermentation processes, producing hydrophilic EPS that substantially enhances the rehydration properties and cooking quality of fat-free instant noodles (FFNs). This improvement is achieved through increased gelatinization and reduced relative crystallinity, which additionally enhances the in vitro starch digestibility of FFNs [51]. Furthermore, *W. confusa* strains W1 and W2 exhibit enhanced adhesion properties and environmental stress resistance attributable to their robust EPS production capabilities, positioning them as suitable candidates for diverse food industry applications [52].

Genomic analysis of *Weissella* species has provided insights into their probiotic potential and safety profiles. Rocha et al. conducted complete genome sequencing of *W. paramesenteroides* UFTM 2.6.1, isolated from unpasteurized milk, followed by comprehensive pangenomic analysis. This investigation identified 99 unique genes associated with probiotic functions, encompassing genes involved in stress response mechanisms, gastrointestinal persistence, and vitamin biosynthesis pathways. In vitro antimicrobial assays confirmed the strain’s activity against Listeria spp. The absence of CRISPR-Cas arrays, Cas proteins, and resistance or virulence genes demonstrated the safety profile of this strain, providing a foundation for *Weissella* applications in food safety and biotechnology [53].

Exopolysaccharides represent complex carbohydrate compounds synthesized during microbial growth and development, functioning as adaptive responses to environmental conditions. These polymers are classified into homopolysaccharides and heteropolysaccharides based on their compositional characteristics. Probiotic bacteria constitute a significant proportion of EPS-producing microorganisms, with *Weissella* species exhibiting exceptional glucan EPS production capabilities. Environmental factors influence both EPS production and bioactivity, with sucrose enhancing overall EPS yield while galactose specifically enhances the anti-inflammatory properties of the resulting polymers [54,55].

The EPSs produced by *Weissella* species are predominantly heteropolysaccharides composed of eight distinct monosaccharides, with glucose and galactose representing the most abundant components [43]. The complex polysaccharide architecture of *Weissella*-derived EPS contributes to their substantial antioxidant activity, particularly through interactions with DPPH radicals. The synergistic effects of antioxidant components and unique glycosidic configurations play crucial roles in preventing oxidative damage in biological systems [56].

The biosynthesis of EPS occurs primarily through extracellular synthesis pathways involving three sequential steps: monosaccharide and sugar nucleotide synthesis, polysaccharide assembly, and transport of synthesized polysaccharides to the extracellular environment [57]. The efficiency and purity of EPS extraction vary considerably depending on the producing strain and isolation methodology employed. Common EPS isolation techniques include precipitation from culture media, alkaline treatment, ion scavenging approaches, and ultrasonication methods [58]. Size-exclusion chromatography represents an efficient approach for separating crude EPS preparations. Purification procedures typically employ protein removal techniques, EPS precipitation methods, and membrane filtration processes. Repeated purification cycles using gel permeation chromatography can yield sufficient quantities of highly purified EPS [59,60]. However, the lack of comprehensive information regarding EPS cellular localization continues to present challenges for developing efficient extraction and purification methodologies.

## 6. *Weissella’s* Association with Health

### 6.1. Weissella and Gut Health

**Figure 2:**
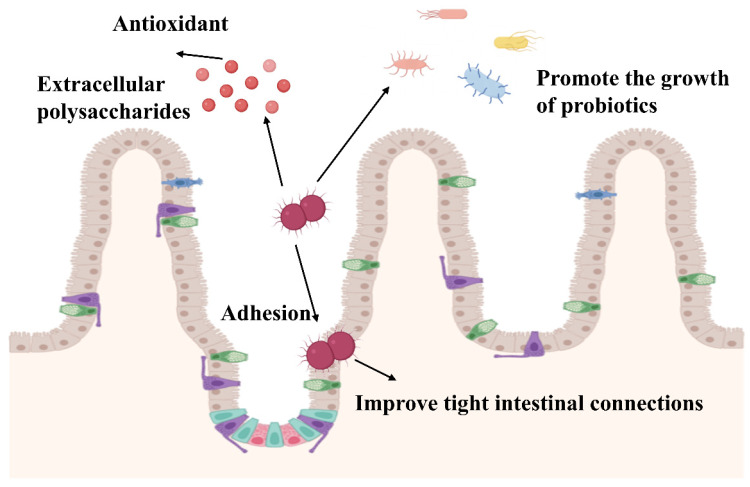
Mechanisms by which *Weissella* promotes intestinal health.

The gut microbiota represents a critical component of human health maintenance, with *Weissella* serving as one of the essential cellulose-degrading genera that contribute significantly to intestinal homeostasis (Figure 2). Specifically, *Weissella* species employ specialized enzymatic systems to degrade indigestible polysaccharides and subsequently produce glucan, which functions within the body’s antioxidant defense system to protect the intestinal barrier and mitigate intestinal damage. For instance, *W. cibaria* produces β-xylosidase enzyme, which hydrolyzes ammonia-pretreated rice straw (A-PRS) to generate bioactive xylooligosaccharides [61]. In addition, after the rainbow trout was fed a plant-based diet for the first time, the intestinal microorganisms changed to Firmicutes, which can metabolize carbohydrates, mainly including *Streptococcus*, *Leuconostoc* and *Weissella* [62,63].

Given that the gut microbiota comprises a complex ecosystem consisting primarily of bacteria, fungi, viruses, and archaea, microbial diversity serves as the fundamental basis for maintaining intestinal health. In this context, research demonstrates that galactan exopolysaccharides produced by *W. confusa* KR780676 promote the growth of beneficial bacteria, specifically enhancing the proliferation of Lactobacillus plantarum MTCC9510 and Lactobacillus fermentum MTCC903 [64]. Similarly, glucans synthesized by *W. cibaria* RBA12, characterized by a 97% α-(1→6) backbone structure with 3% α-(1→3) branching, stimulate the in vitro growth of Bifidobacterium and Lactobacillus species [65]. Collectively, these findings indicate that *Weissella* species support gut health through mechanisms that increase both the abundance and diversity of beneficial bacterial populations.

Beyond promoting beneficial bacterial growth, specific polysaccharide components produced by *Weissella* species demonstrate distinct protective mechanisms for intestinal barrier function. Notably, Zhao et al. isolated a polysaccharide component designated EPS-2 with a molecular weight of 845 kDa from *W. cibaria*. In contrast to other exopolysaccharides, EPS-2 exhibits direct protective effects on the intestinal barrier by reversing the propionate level decreases associated with colitis, thereby improving colonic goblet cell function and mucin content. Furthermore, mechanistic studies reveal that propionate binds to G protein-coupled receptor 43 on intestinal epithelial cell surfaces, consequently increasing histone acetylation levels, promoting expression of tight junction proteins occludin and ZO-1, and enhancing mucin production [66,67].

In addition to barrier protection, *Weissella* species demonstrate substantial capacity for alleviating oxidative stress within the intestinal environment, consequently reducing oxidative damage. The exopolysaccharides produced by these bacteria exhibit exceptional antioxidant activity, which represents a key mechanism underlying their protective effects [42,68]. More specifically, during lipopolysaccharide (LPS) exposure, *W. cibaria* MW01 inhibits nuclear translocation of NF-κB and inactivates the myosin light chain kinase MLCK-pMLC pathway, thereby attenuating the secretion of pro-inflammatory cytokines including TNF-α and IL-6, and mitigating intestinal inflammation [69].

Moreover, the regulatory mechanisms governing NF-κB activity involve SIRT1-mediated pathways. Particularly, SIRT1 activation induces deacetylation of NF-κB-p65 at lysine 310 (K310) and histone H3 at lysine 9 (K9). Consequently, Sirtuin1 (SIRT1) activation results in reduced NF-κB-p65 DNA binding capacity and mitigates oxidative stress through decreased transcription of NADPH oxidase subunits [70]. Supporting these mechanisms, in DSS-induced mouse models of intestinal inflammation, *W. paramesenteroides* NRIC1542 increased SIRT1 protein expression, suppressed NF-κB activation, and reduced TNF-α and IL-1β levels [71].

The antioxidant properties of *Weissella* species have been further validated through studies utilizing alternative model systems. Specifically, the antioxidant effects were demonstrated in the *Caenorhabditis elegans* model system, where fructan produced by *W. cibaria* MD2 enhanced oxidative stress resistance and extended lifespan in *C. elegans* through promotion of nuclear translocation of the transcription factor DAF-16 [72].

Finally, *Weissella* species demonstrate direct adhesive interactions with intestinal tissues to exert protective functions. Remarkably, in gut barrier dysfunction models, *W. cibaria* MW01 exhibited superior adhesion to intestinal cells compared with *Lactobacillus rhamnosus* GG, while simultaneously mitigating LPS-induced inflammation, preventing tight junction protein downregulation, and restoring gut barrier integrity [69]. Similarly, in the hydrogen peroxide-induced inflammatory bowel disease model, *W. confusa* F213 could increase the transmembrane epithelial resistance and ZO-1 expression to maintain mucosal integrity [73]. These multifaceted protective mechanisms underscore the significant potential of *Weissella* species in maintaining and restoring intestinal health.

### 6.2. Weissella and Other Diseases

The probiotic properties of *Weissella* extend beyond gut health, encompassing reported benefits in obesity prevention, inflammation modulation, and detoxification processes. These diverse therapeutic applications demonstrate the multifaceted potential of *Weissella* species in health promotion and disease prevention (Table 2). Regarding obesity prevention, *W. koreensis* OK1-6 inhibits fat accumulation in 3T3-L1 adipocytes through downregulation of key lipogenic genes, including CCAAT enhancer-binding protein α (C/EBPα), sterol regulatory element binding protein 1 (SREBP1), and fatty acid synthase (FAS), thereby indicating its potential in preventing obesity development [74]. Complementing these findings, *W. cibaria* MG5285 suppresses lipogenic protein expression and promotes phosphorylation of adenosine 5′-monophosphate (AMP)-activated protein kinase (AMPK) and acetyl-CoA carboxylase in hepatic tissue, consequently reducing lipid accumulation [75]. Collectively, these findings highlight the capacity of *Weissella* species to inhibit fat synthesis pathways, thereby driving further investigation into their potential as next-generation anti-obesity probiotics. In addition to metabolic regulation, *Weissella* species exhibit significant anti-inflammatory properties through multiple mechanisms. In vitro studies have demonstrated that *W. cibaria* strains alleviate lipopolysaccharide (LPS)-induced inflammatory responses by reducing the production of nitric oxide, IL-6, and IL-1β in macrophage cultures [76]. Furthermore, in dermatological inflammation models, oral administration of *W. cibaria* WIKIM28 effectively reduced atopic dermatitis-like skin lesions, epidermal thickening, and serum immunoglobulin E (IgE) levels [77].

To further elucidate the anti-inflammatory mechanisms of *Weissella*, Hong et al. isolated lipoteichoic acid from *W. cibaria* and reported that this compound promoted immune factor secretion in a dose-dependent manner. Additionally, lipoteichoic acid activates NF-κB, p38, and c-Jun N-terminal kinase phosphorylation in THP-1 cells while simultaneously stimulating immune factor secretion in mouse splenocytes, suggesting potential therapeutic applications in treating immunosuppressive diseases [78]. Aging could cause chronic inflammation. Surprisingly, compared with middle-aged people, the abundance of *Weissella* in intestinal microorganisms in the elderly was significantly increased, which suggested that the anti-inflammatory effect of *Weissella* might be achieved by compensatory increase in its abundance [79]. In Alzheimer’s disease, microglia and astrocytes are involved in its inflammatory response [80]. Studies have shown that *Weisslla* can reduce oxidative stress and reduce cognitive impairment of Alzheimer’s disease through SIRT1/PGC-1α [81]. Beyond metabolic and immunological benefits, *Weissella* species also demonstrate significant potential in detoxification processes, particularly for heavy metal remediation. The liver and kidneys, which serve as critical organs for heavy metal metabolism, are particularly vulnerable to heavy metal toxicity. In vivo investigations have shown that *W. cibaria* WD2 effectively alleviates cadmium- and lead-induced hepatic and renal damage [82]. Mechanistically, in vitro studies revealed that W. viridescens ZY-6 adsorbs cadmium ions (Cd^2+^) primarily through electrostatic interactions with negatively charged functional groups, including hydroxyl (-OH), amino (-NH_2_), carboxylate (COO^−^), and phosphoryl (P=O) groups present on the bacterial cell surface. Supporting these findings, scanning electron microscopy and Fourier transform infrared spectroscopy confirmed that Cd^2+^ adsorption induces physiological changes in bacterial cells, characterized by cellular wrinkling and elongation [83]. These observations suggest that *Weissella* species can function as effective biosorbents for mitigating cadmium contamination in environmental systems, human dietary sources, and animal feed applications.

**Table 2:** Probiotic characteristics and applications of *Weissella*.

Strain	Probiotic Properties	Application	References
*W. confusa* WM36	Produce antimicrobial substances such as antimicrobial peptides, organic acids, and 2,4-di-tert-butylphenol	Alternative therapies as non-antibiotic approaches for typhoid fever control	[47]
*W. cibaria* CMS1	Inhibit the formation of the Streptococcus mutans biofilm	Decrease the risk of respiratory tract and intestinal infections	[84]
*W. cibaria* WIKIM28	Inhibit local accumulation and degranulation rate of mast cells	Potential use as a dietary supplement or therapeutic agent	[77]
*W. confusa* PL9001	Target and destroy bacterial cell wall by secreting bacteriocin with bactericidal activity	Development of a novel gastric probiotic	[85]
*W. paramesenteroides* MYPS5.1	Product high concentrations of extracellular polysaccharides	Potential anticancer agent	[86]
*W. confusa* F213	Enhance the resistance of the gastrointestinal environment	Adjuvant therapy for inflammatory bowel diseases	[73]
*W. viridescens* UCO-SMC3	Produce lactic acid, hydrogen peroxide, and bacteriocins with strong bactericidal activity	Reduce inflammatory response.	[87]
*W. cibaria* CMU	Produce bactericidal substances: hydrogen peroxide and organic acids (lactic acid, acetic acid, and citric acid)	Improve oral health and prevent oral diseases	[88]
*W. sp.* SNUL2	Produce peptidases	Regulate gut microbiota composition	[89]
*W. viridescens* Wv2365	Produce exopolysaccharides	Improve symptoms of metabolic dysfunction-associated steatotic liver disease	[90]

## 7. Challenges and Future Research Directions

Despite significant advances in *Weissella* research, several critical challenges and limitations continue to impede comprehensive understanding and practical application of these microorganisms. Although whole-genome sequencing, metabolomics, and isotope tracing techniques have provided valuable insights into the metabolic pathways of *Weissella*, their application remains constrained by methodological limitations. For instance, metagenomics approaches may not adequately distinguish functional differences between closely related strains, thereby necessitating more refined genomic and metabolomic analytical approaches to elucidate strain-specific characteristics and capabilities. Furthermore, while exopolysaccharides represent the most extensively studied metabolites of *Weissella*, other potentially significant metabolites, particularly short-chain fatty acids (SCFAs), have received comparatively limited research attention. Consequently, the structural and functional diversity of bioactive compounds produced by *Weissella* species, as well as their specific roles in different environmental contexts, require comprehensive investigation to fully understand their therapeutic and industrial potential.

Notably, a promising characteristic of *Weissella* species is their demonstrated tolerance to gastric acid and bile salts [64,91], a trait that significantly enhances their potential for broader industrial and therapeutic applications. However, despite the well-documented probiotic properties of most *Weissella* strains, their potential pathogenicity cannot be overlooked and represents a critical safety concern. Specifically, *W. ceti* has been reported to cause mortality rates reaching 60% in farmed rainbow trout populations in Peru, highlighting the substantial risks associated with insufficient understanding of the pathogenic potential within the *Weissella* genus [92]. Nevertheless, current safety evaluations of *Weissella* species have yielded promising results, particularly in mammalian model systems. Studies conducted in rat models have demonstrated that *W. cibaria* exhibits safety at doses as high as 5000 mg/kg body weight (bw)/day (1.8 × 10^9^ CFU/kg bw/day), with no evidence of mutagenicity or chromosomal aberrations [93]. Additionally, phenotypic safety assessments of *W. cibaria* JW15 have not detected the presence of virulence genes, including cytolysin (*cylA*), aggregation substance (*asa1*), hyaluronidase (*hyl*), and gelatinase (*gelE*) [94]. Moreover, the exopolysaccharides produced by *Weissella* bacteria have been granted generally recognized as safe status, supporting their potential for food industry applications [55]. Despite these encouraging safety profiles in animal models, *Weissella* research still lacks adequate clinical validation in human populations, representing a significant gap in translational research. Furthermore, in view of the resistance to vancomycin of *Weisseria* isolated from different environments, comprehensive safety evaluations are still required, particularly regarding quorum-sensing systems and long-term health effects in human hosts [95]. Even if *Weissella* species receive regulatory approval as probiotics, substantial research efforts will be necessary to address remaining challenges related to encapsulation technologies, the safety and efficacy profiles of their metabolites, and their long-term impact on host health dynamics. These multifaceted research and development efforts will be critical to fully harness the therapeutic and industrial potential of *Weissella* species while ensuring their safe and effective application in both commercial and clinical settings. Addressing these challenges through systematic investigation will be essential for advancing *Weissella* from promising research candidates to validated therapeutic and industrial agents.

## 8. Conclusions

This comprehensive review demonstrates that *Weissella* represents a versatile genus of lactic acid bacteria with significant potential for human health and industrial applications. The research reveals *Weissella*’s multifaceted nature, encompassing probiotic properties, antimicrobial activities, and biotechnological applications, particularly in exopolysaccharide production. The genus produces diverse bioactive compounds including bacteriocins, organic acids, and EPS that contribute to antioxidant, anti-inflammatory, and prebiotic functions while promoting gut health through enhancing beneficial bacterial growth, strengthening intestinal barrier function, and modulating immune responses. However, this review highlights the dual nature of *Weissella*, with certain strains like *W. confusa* exhibiting opportunistic pathogenic behavior, underscoring the critical importance of strain-specific characterization and safety assessments before clinical applications. Future research should prioritize multi-omics approaches to understand strain-specific differences and identify genetic markers distinguishing beneficial from pathogenic strains. Critical areas include elucidating molecular mechanisms of host-microbe interactions, conducting long-term clinical trials to establish safety profiles and therapeutic efficacy, optimizing industrial cultivation methods and encapsulation technologies, developing regulatory frameworks for safety assessment and quality control, and exploring environmental applications in bioremediation and agriculture. The remarkable potential of *Weissella* warrants continued investment in research and development, promising innovative solutions for human health, food technology, and sustainable biotechnological applications.

## Data Availability

No new data were created or analyzed in this study. Data Sharing is not applicable to this article.

## Abbreviations

The following abbreviations are used in this manuscript:

AMPK	adenosine 5′-monophosphate (AMP)-activated protein kinase
A-PRS	ammonia-pretreated rice straw
CCAAT	CCAAT enhancer-binding protein α
EPS	exopolysaccharides
FAS	fatty acid synthase
FFNs	fat-free instant noodles
IgE	immunoglobulin E
RSM	Response Surface Methodology
SCFAs	short-chain fatty acids
SIRT1	Sirtuin1
SREBP1	sterol regulatory element binding protein 1
